# Primary cardiac intimal sarcoma masquerading as mitral stenosis

**DOI:** 10.1002/ccr3.1089

**Published:** 2017-07-14

**Authors:** Michael Spartalis, Eleni Tzatzaki, Eleftherios Spartalis, Demetrios Moris, Antonios Athanasiou, Stamatios Kyrzopoulos, Dimitrios Tsiapras, Vassilis Voudris

**Affiliations:** ^1^ Division of Cardiology Onassis Cardiac Surgery Center Athens Greece; ^2^ Laboratory of Experimental Surgery and Surgical Research Medical School University of Athens Athens Greece; ^3^ Department of Surgery The Ohio State University Comprehensive Cancer Center The Ohio State University Columbus Ohio; ^4^ Department of Surgery Mercy University Hospital Cork Ireland

**Keywords:** Cardiac tumors, mitral stenosis, sarcoma

## Abstract

Intimal sarcomas are a very rare medical entity and usually arise from the pulmonary artery or the thoracic aorta. Sarcomas should be considered in the differential diagnosis in patients with suspected mitral valve disease. Echocardiography should be performed as early as possible to establish a prompt diagnosis and management.

## Case Presentation

A 41‐year‐old woman with no previous medical history presented with exertional dyspnea, orthopnea, and fatigue. The patient had a blood pressure of 100/60 mmHg. Electrocardiography showed sinus tachycardia (110 beats/min). A systolic murmur was present at the apex, and the chest X‐ray revealed pulmonary congestion and bilateral pleural effusion in keeping with acute pulmonary edema.

**Figure 1:**
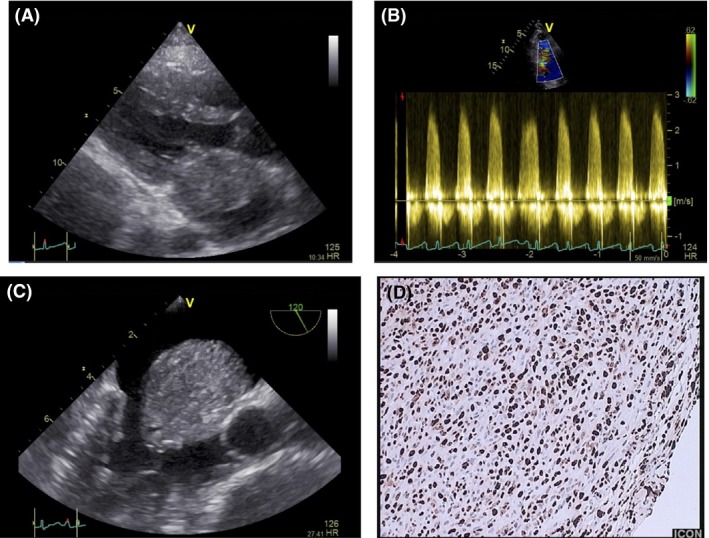
(A) Parasternal long‐axis view of transthoracic echocardiography (TTE): Large left atrial mass. (B) TTE continuous wave Doppler: large left atrial mass causing mitral obstruction (mean pressure gradient: 16 mmHg). (C). Transesophageal echocardiography (TEE): large mass occupying the entire atrial cavity. (D). Hematoxylin and eosin staining method: all tumor cells in cycling phase.

Transthoracic echocardiography revealed a left atrial tumor causing a severe mitral obstruction (mean pressure gradient: 16 mmHg), leading to severe pulmonary hypertension (right ventricular systolic pressure: 55 mmHg) with preserved left ventricular ejection fraction (Fig. 1A and B). Transesophageal echocardiography revealed a large mass occupying the entire atrial cavity, extending to the posterior leaflet of the mitral valve and the left atrial appendage (Fig. 1C). The patient underwent an urgent surgery under cardiopulmonary bypass. As the tumor seemed to extend over most of the surface of the endocardium, mitral annulus reconstruction was impossible. The patient was subjected to mitral valve replacement and radiofrequency (RF) ablation to the site of the tumor, in order to prevent a recurrence of the tumor.

Histopathology showed large poorly differentiated epithelioid and pleomorphic cells infiltrating the myocardial tissue. Hematoxylin and eosin staining revealed that all tumor cells were in cycling phase (Fig. 1D). The resected tumor was consistent with an intimal sarcoma.

The patient underwent radiotherapy. Nine months later, follow‐up echocardiography has detected no signs of recurrence.

## Authorship

MS and ET: involved in conception and design of the research and writing of the manuscript; ET, SK: involved in acquisition of data; ES, DM, and AA: involved in analysis and interpretation of the data; DT and VV: involved in critical revision of the manuscript for intellectual content.

## Conflicts of Interest

The authors report no financial relationships or conflicts of interest regarding the content herein.

